# Insights into the mechanisms, regulation, and therapeutic implications of extracellular matrix stiffness in cancer

**DOI:** 10.1002/btm2.10698

**Published:** 2024-07-31

**Authors:** Ximo Zhang, Abdullah Al‐Danakh, Xinqing Zhu, Dan Feng, Linlin Yang, Haotian Wu, Yingying Li, Shujing Wang, Qiwei Chen, Deyong Yang

**Affiliations:** ^1^ Department of Urology First Affiliated Hospital of Dalian Medical University Dalian China; ^2^ Department of Discipline Construction Dalian Medical University Dalian China; ^3^ Department of Biochemistry and Molecular Biology, Institute of Glycobiology Dalian Medical University Dalian China; ^4^ Zhongda Hospital, Medical School Advanced Institute Life Health Southeast University Nanjing China; ^5^ Department of Surgery Healinghands Clinic Dalian China

**Keywords:** cancer, ECM stiffness, extracellular matrix, signaling pathways, targeted therapy, tumor microenvironment

## Abstract

The tumor microenvironment (TME) is critical for cancer initiation, growth, metastasis, and therapeutic resistance. The extracellular matrix (ECM) is a significant tumor component that serves various functions, including mechanical support, TME regulation, and signal molecule generation. The quantity and cross‐linking status of ECM components are crucial factors in tumor development, as they determine tissue stiffness and the interaction between stiff TME and cancer cells, resulting in aberrant mechanotransduction, proliferation, migration, invasion, angiogenesis, immune evasion, and treatment resistance. Therefore, broad knowledge of ECM dysregulation in the TME might aid in developing innovative cancer therapies. This review summarized the available information on major ECM components, their functions, factors that increase and decrease matrix stiffness, and related signaling pathways that interplay between cancer cells and the ECM in TME. Moreover, mechanotransduction alters during tumorogenesis, and current drug therapy based on ECM as targets, as well as future efforts in ECM and cancer, are also discussed.

**Translational Impact Statement**

This article provides an overview of the intricate interplay between stromal mechanisms and the processes of tumorigenesis and cancer progression. Matrix mechanics have emerged as a crucial factor influencing cancer progression, and this article highlights its mechanisms of action. Furthermore, the article delves into drugs aimed at modulating the mechanical aspects of the matrix to offer hope for improved cancer treatments. However, it is worth noting that many of these treatments are still in the preclinical or clinical trial phases, awaiting further validation by the scientific community.

## 1 INTRODUCTION

The incidence and mortality rates of cancer are increasing over time. According to the most recent data available for 2022, it is projected that the United States will experience 1,918,030 new cases of cancer, with 609,360 deaths attributed to the disease.^1^ As a result, cancer is quickly becoming one of the deadliest diseases threatening the general population's health. The most apparent hallmarks of cancer are the uncontrolled growth of cancer cells, their local invasion, and their potential to spread throughout the body. Surgical, chemotherapeutic, radiotherapeutic, targeted, and immunotherapeutic treatments are typical cancer treatments today.^2^ However, many alternatives are starting to exist, like stem cell therapy, ablation therapy, and so forth.^3^, ^4^ Recurrent tumors and distant metastases are the most common causes of cancer‐related mortality following systemic treatment of the initial tumor. Although great advances have been achieved in cancer treatment in recent years, particularly in targeted therapy and immune therapy, the effort to transform this life‐threatening disease into a chronic, manageable condition continues. When developing breakthrough cancer treatments, it is equally crucial to have a comprehensive understanding of cancer cells as well as the environment that promotes their malignant behavior.

**FIGURE 1:**
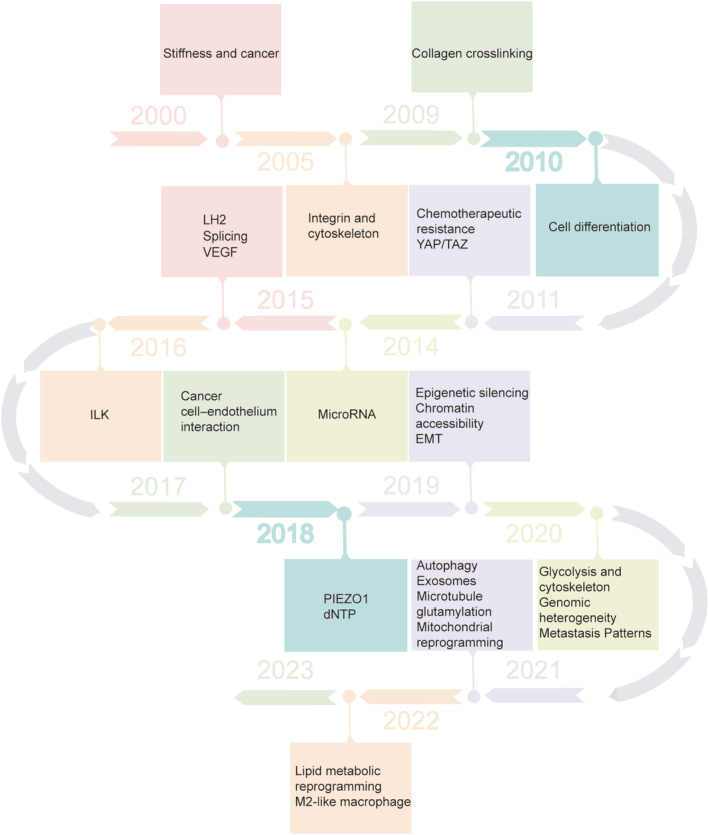
Timeline of extracellular matrix (ECM) stiffness development. (YAP/TAZ, Yes‐associated protein and Transcriptional co‐activator with PDZ‐binding motif; LH2, Lysyl hydroxylase 2; EMT, Epithelial‐Mesenchymal Transition; ILK, Integrin‐Linked Kinase; PIEZO1, Piezo‐type Mechanosensitive Ion Channel Component 1).

Through mutual and dynamic crosstalk, the rearrangement of the tumor microenvironment (TME) components significantly impacts tumorigenesis and progression. TME comprises multiple cell types, including tumor cells, stromal fibroblasts, endothelial cells, microglia, macrophages, lymphocytes, and non‐cellular components of the extracellular matrix (ECM), such as collagen, fibronectin, hyaluronan, and laminin, among others.^5^ The ECM, which consists of interstitial elements found in the tissues or organs of all multicellular organisms, is involved in all physiological processes.^6^, ^7^ It accomplishes this by supplying structural support, anchoring for cell adhesion, a range of growth stimulants, and activation of intracellular signaling pathways. In addition to physical support for tissue integrity and resilience, the ECM dynamic structure is continuously remodeled to control tissue homeostasis, which regulates the biochemical and biomechanical properties of the ECM.^8^ The ECM is characterized by its biochemical composition (including intermolecular covalent cross‐linking) and its biophysical parameters, such as morphology, molecular density, stiffness/rigidity, and tension.^9^ The ECM comprises about 300 proteins, including collagen, proteoglycans (PG), Heparan sulfate proteoglycans (PGs), glycoproteins, and others.^10^ The study of the relationship between stiffness and tumors is a relatively new research field,^11^ but it has attracted an enormous amount of interest and advanced significantly over the past few decades (Figure 1).^11^, ^12^, ^13^, ^14^, ^15^, ^16^, ^17^, ^18^, ^19^, ^20^, ^21^, ^22^, ^23^, ^24^, ^25^, ^26^, ^27^, ^28^, ^29^, ^30^, ^31^, ^32^, ^33^, ^34^ Therefore, a comprehensive understanding of extracellular matrix composition and structural characteristics can facilitate deeper knowledge of the relationship between matrix stiffness and tumors.

The degree to which a substance can withstand force without undergoing significant deformation is called its stiffness.^35^ The amount of ECM present in the tumor, particularly collagen, elastin, and hyaluronic acid, is the primary factor that determines how stiff the tumor is.^32^ Collagen cross‐linking was an important regulator of fiber hyperplasia, and lysyl oxidase (LOX) was responsible for an increase in fibrillary collagen and the stiffness of the ECM.^36^, ^37^ Hypoxia‐inducible factor 1 (HIF‐1) may cause fibrosis through the expression of the LOX gene and promote epithelial–mesenchymal transition (EMT).^38^ Cell contractile forces can also align and deform collagen fibers, thereby stiffening the ECM.^39^, ^40^ In tumors, cancer‐associated fibroblasts (CAFs) increase tumor stiffness through hyperplasia.^40^ This phenomenon is driven by the excessive buildup of fibrillary extracellular matrix proteins, resembling a misdirected or dysregulated wound healing process.^41^ Calponin 1 can increase CAFs‐mediated matrix stiffness in gastric cancer.^42^ Hyaluronic acid (HA) frequently increases in cancer via distinct classes of integrins that either amplify or overcome mechanical signals to produce cellular phenotypes that can only be seen on stiff ECM.^43^ The stiffness of the collagen network increased with HA molecular weight and concentration, with high HA molecular weight reducing migration and increasing collagen network contraction.^44^ Matrix metalloproteinases (MMPs) are the main enzymes that degrade ECM and hence play a vital role in the dynamic and structure of the cancer microenvironment. Tissue inhibitors of metalloproteinases (TIMP) are important for tissue integrity, and the ratio of MMP to TIMP determines the overall proteolytic activity.^45^ Another study has demonstrated that epigenetic silencing of the tumor suppressor RASSF1A can contribute to an increase in ECM stiffness. Specifically, lung cancer cells with RASSF1A promoter methylation exhibited constitutive nuclear YES‐associated protein 1 (YAP1) accumulation and upregulated expression of prolyl 4‐hydroxylase alpha‐2 (P4HA2), which led to increased ECM stiffness via mediation of collagen deposition.^23^ Another crucial aspect to address involves the non‐elastic characteristics of the ECM, allowing for its irreversible remodeling by dynamic mechanical forces generated by cells.^46^ Consequently, ECM remodeling is not solely driven by the deposition and degradation of ECM but is also influenced by mechanical forces.^47^ Cells exert dynamic forces on the ECM through protrusions, inducing local densification of the ECM, resulting in locally fibrotic regions, and this form of ECM remodeling is facilitated by the non‐elastic or viscoplastic properties of the ECM. Viscoplasticity enables the ECM material to respond differently to forces compared to elastic materials, which deform and recover after force relaxation. This property may lead to the softening of the ECM over time, even when initially stiff, due to applied forces. Additionally, spatial stiffness profiles can change due to non‐elasticity, with regions experiencing increased ECM densification becoming stiffer through force‐induced cell remodeling. Molecularly, viscoplasticity in the ECM is attributed to molecular bonds that can reorganize or break under applied forces and play a crucial role in cell spreading and differentiation.^48^

## 2 ROLE OF ECM STIFFNESS IN CANCER

**FIGURE 2:**
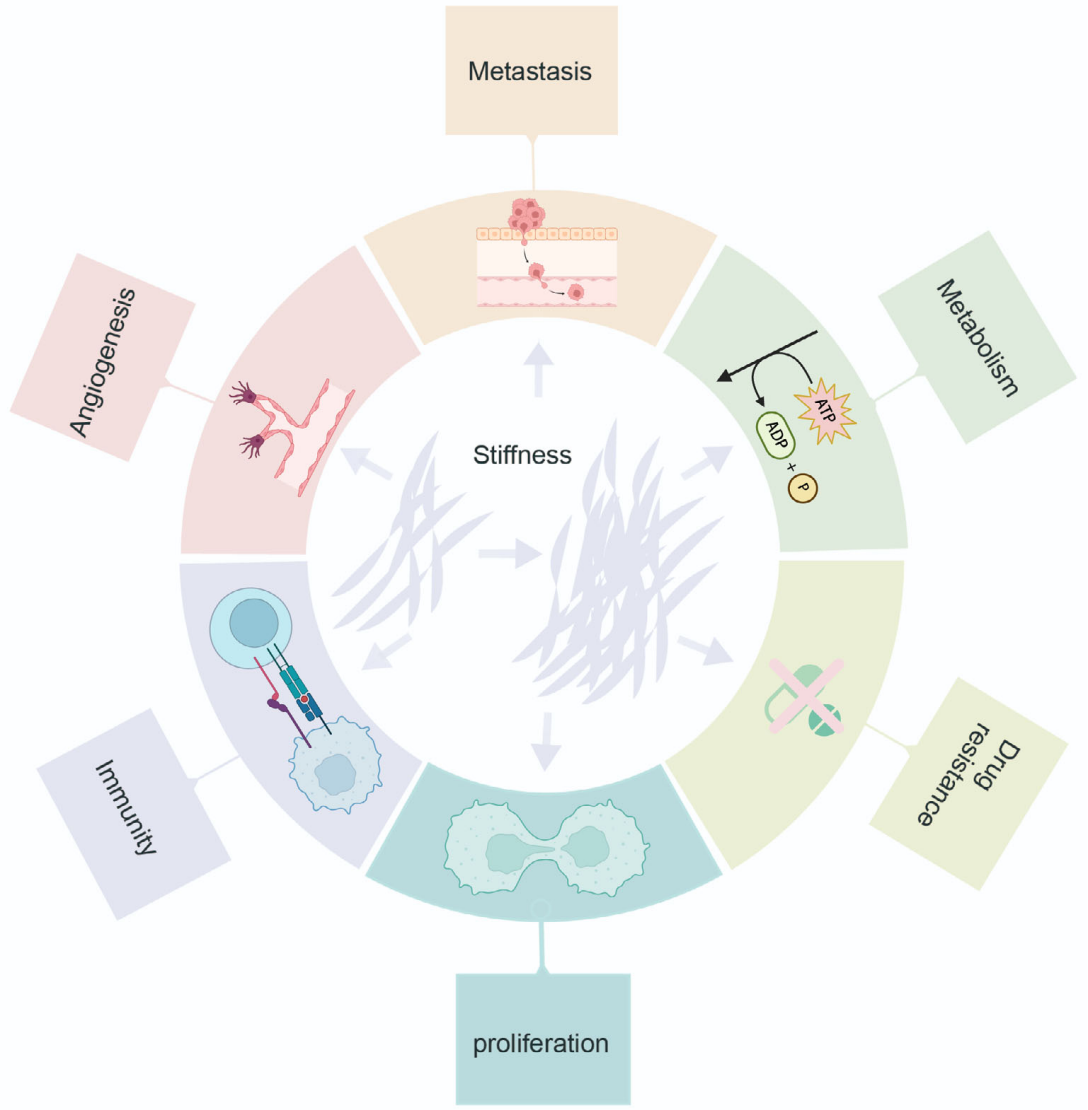
The oversight of crucial mechanisms of ECM, such as tumor angiogenesis, growth, metastasis, metabolism, proliferation, immune evasion, and drug resistance, through alterations in the stiffness of the ECM. The augmentation in the stiffness of the ECM results in the production of mechanical cues that instigate diverse cellular reactions.

ECM stiffness plays a multifaceted role in regulating crucial aspects of cancer progression. Understanding how ECM stiffness influences proliferation and metastasis, drug resistance, angiogenesis, metabolism, and immunity evasion (Figure 2) is vital for developing effective therapeutic strategies that target the ECM and its associated signaling pathways. By unraveling the complex interplay between cancer cells and their mechanical microenvironment, we can pave the way for novel approaches to improve cancer treatment outcomes and patient survival.

### 2.1 ECM stiffness role in the regulation of proliferation

The morphology of cancer cells has been linked to their proliferative capability.^49^ Enlarged cell dimensions and the presence of pseudopodia are believed to enhance nutrient absorption, thus fostering cell proliferation. The integration of signaling pathways from soluble mediators and the ECM occurs within the cytoskeleton.^50^ Tension within the cytoskeleton can either suppress or activate various growth factor signaling pathways, consequently inhibiting or promoting mitogenic responses to ECM physical stimuli.^13^ The current findings indicate that matrix stiffness influences cell proliferation through mechanisms beyond cell morphology and spreading area.^51^, ^52^ It likely interacts with other regulatory factors affecting proliferation in both normal and cancer cells in which the ECM plays a vital role in maintaining the structural and mechanical integrity of tumor tissues. Increased ECM stiffness, including collagen deposition, promotes focal adhesion assembly and enhances cytoskeletal functions in cancer cells, ultimately supporting their proliferation.^50^, ^51^, ^52^ Cirrhosis of the liver is well‐documented as a significant risk factor for the carcinogenesis of liver cancer. Research employing matrix stiffness levels of 1, 6, and 12 kPa to simulate normal, fibrotic, and cirrhotic hepatocellular carcinoma (HCC) tissue, respectively, demonstrated a 2.7‐fold and 12.2‐fold increase in Ki‐67 staining in Huh7 and HepG2 cells, respectively, when cultured on a 12 kPa substrate compared to a 1 kPa substrate. Additionally, the expression levels of proliferating cell nuclear antigen (PCNA) and Cyclin D1, along with cell numbers, exhibited similar trends in response to increased matrix stiffness in both cell lines. Enhanced phosphorylation of focal adhesion kinase (FAK), extracellular signal‐regulated kinase (ERK), protein kinase B (PKB/AKT), and signal transducer and activator of transcription 3 (STAT3) on the 12 kPa substrate indicates that matrix stiffness regulates cell proliferation through multiple signaling pathways.^13^, ^50^ This study confirmed the biomechanical composition of the ECM significantly influences the proliferative capacity of HCC cells. Consistently, other studies have shown that increased matrix stiffness enhances AKT activity via the oncogene ZNF217, activating the PI3K/Rac and ERK pathways to promote cell proliferation and invasion.^53^ Piezo proteins, which sense mechanical signals and promote tissue stiffening, also modulate cell proliferative capacity by activating the AKT/mTOR pathway.^54^ Recent findings indicate that C‐X‐C chemokine receptor type 4 (CXCR4) is a key mediator in the interaction between matrix stiffness signaling and the molecular switch, controlling HCC cell growth through the YAP signaling pathway.^55^ Another observations were made with SKOV‐3 cells cultured on substrates of varying stiffness (0.5, 4, and 25 kPa), with increased cell proliferation rates and YAP expression associated with stiffer matrices.^56^ Activation of YAP/TAZ signaling leads to nuclear accumulation, stimulating tumor cell proliferation by modulating cell cycle, DNA replication, repair, and mitosis.^57^ Mechanical signals, for example, can change the cell cycle, promote G1/S transition in human hepatoma HepG2 cells, and regulate cell proliferation.^58^ ECM conformity‐dependent integrin clustering regulates growth factor‐dependent extracellular signal‐regulated kinase (ERK) activation by affecting Rho‐generated cytoskeletal tension and focal adhesion (FA) formation, thereby controlling cell growth and tissue behavior.^59^ In osteosarcoma cells (U‐2 OS and MG‐63), higher substrate stiffness resulted in increased proliferative indices and Ki‐67 expression, while softer substrates enhanced self‐renewal, differentiation potential, and drug resistance, highlighting the dual role of ECM stiffness in tumor growth and metastasis.^60^ ECM stiffness caused by increased collagen cross‐linking promotes breast malignancy by enhancing integrin‐growth factor receptor crosstalk.^36^ Furthermore, regulation of the ILK (integrin‐linked kinase) by ECM stiffness has been found to facilitate the development of breast cancer stem cells (CSCs) and support tumor formation and metastasis.^19^ On the other hand, increased ECM stiffness has been shown to hinder proliferation in neuroblastoma and reduce N‐Myc expression, which plays a role in neuroblastoma differentiation.^61^

### 2.2 ECM stiffness role in the regulation of metastasis

The ECM harbors intricate local structures and architectures that substantially influence cell behaviors, encompassing migration and differentiation. These encompass nanotopographical cues like fiber alignment and mesoscopic structures such as dense collagen clusters.^62^, ^63^, ^64^, ^65^ Notably, mesoscopic architectures, including dense collagen clusters, can significantly affect cell migration and differentiation. These ECM attributes and their impacts extend beyond overall stiffness, contributing to heterogeneous and anisotropic stiffness profiles at the cellular level. Furthermore, ECMs characterized by global softness may exhibit local stiffness (attributed to mesoscopic architectures), emphasizing the need to consider spatial scales in the conceptualization of stiffness.^66^, ^67^

Tumor cells on high‐stiffness ECM show higher actin expression,^20^ form prominent stress fibers that enable them for FAs, and migrate rapidly.^52^ In addition to the chemical gradient, or chemotaxis, the collective directional migration of cells also follows the stiffness gradient, or durotaxis,^68^ and moves in the direction of the high‐stiffness microenvironment. It is believed that FA is the molecular mechanism responsible for the movement, guiding coordinated cell migration.^36^

A team of scientists investigated cancer's mechanical guidance processes and surroundings. Their findings indicate that mechanical guidance occurs largely in mesenchymal cancer cells and not in epithelial cancer cells, hinting that cell contractility may play a role in anisotropic stiffness gradient‐regulated cell migration.^69^ Another study found that the orientation of collagen fibers inside the tumor microenvironment directs tumor cell migration.^70^ Collagen alignment improves migration efficiency by increasing migration distance.^71^ One of the important regulators for collage is lysyl hydroxylase 2 (LOXL2), which has been found to cause a transition in collagen cross‐linking in tumor stroma.^72^ Increased ECM stiffness enhances LOXL2 expression, which in turn stimulates fibronectin production, MMP9, and CXC chemokine ligand 12 (CXCL12) expression, and the recruitment of bone marrow‐derived dendritic cells to aid in the development of pre‐metastatic niches.^73^ Previous studies proved that the stiffness of the ECM directly contributes to an increase in the number of invadopodia as well as their activity.^74^ Invadopodia are actin‐rich subcellular processes cancer cells exploit with associated proteases to destroy the ECM.^75^ High‐stiffness cells have been shown to have increased expression of Mena, a protein related to invasive invadopodia and breast cancer metastasis.^76^ A stiff ECM can also induce mechanotransduction linker srGAP2 tension gradients to control migration direction in triple‐negative breast cancer (TNBC).^77^ Prostate cancer exhibits varied metastatic patterns based on the stiffness of the ECM, with high stiffness inducing YAP/PDZ‐binding protein (TAZ) nuclear localization and promoting cell migration and low stiffness promoting cell migration via the upregulation of CD44 expression.^78^

ECM stiffness independently regulates the switching of cell responses to transforming growth factor (TGF‐β1) between EMT and apoptosis. Tissues with decreased stiffness are more susceptible to TGF‐β1‐induced apoptosis; however, tissues with higher stiffness are more likely to undergo EMT, resulting in more collagen, fibronectin, and procollagen lysyl hydroxylase.^79^ In breast cancer, increased ECM stiffness modulates EMT and metastasis by mechanically sensitive EPHA2/LYN protein complexes or by releasing the mechanical medium TWIST1 from its cytoplasmic binding partner G3BP2.^80^, ^81^ Studies on hepatocellular carcinoma (HCC) demonstrate that an increase in hepatic stiffness stimulates hepatic stellate cells (HSCs), causing them to develop into myofibroblasts, which causes an increase in HCC ECM contents.^82^ Three signaling pathways independently participated in the stiffness‐mediated effect on EMT, including integrin‐mediated S100A11 membrane translocation, eIF4E phosphorylation, and TGF‐β1 autocrine.^25^ Studies support the belief that keratin downregulation observed during EMT directly contributes to tumor cell migration and invasion behavior.^83^ The presence of tumor‐associated macrophages and ECM stiffness contribute to an aggressive phenotype and regulate the expression of key EMT‐related markers.^51^

Stiffening of the ECM enhances phosphatidylinositol 3‐kinase (PI3K) activity, which regulates tumor invasion.^36^ Exosomal thrombospondin‐1 (THBS1) mediates its stiffness‐dependent pro‐invasive effects by binding MMPs and adhesion kinases.^28^ The ECM stiffens to rewire glutamine (Gln) metabolism to promote microtubule (MT) glutamylation and force MT stabilization, thereby promoting cell invasion.^30^ Stearoyl‐CoA desaturase 1 (SCD1) changes the composition of membrane phospholipids by reprogramming lipid metabolism and increasing cell membrane fluidity, thereby promoting cancer cell invasion and metastasis.^32^ Isocitrate dehydrogenase 1‐mutant glioma can lower ECM stiffness by reducing the abundance of tenascin C in the ECM.^84^

Basement membrane (BM) is a special type of ECM, and netrin‐4 (Net4) is a crucial regulator of BM stiffness. The ratio of Net4 to laminin molecules determines the stiffness of BM, such that the more Net4, the softer the BM, thereby reducing cancer cell invasion activity.^85^

### 2.3 ECM stiffness promotes cancer drug resistance

Increasing ECM stiffness can promote resistance to chemotherapy and immunotherapy in some tumors.^13^ First, the stiffening ECM forms a physical barrier to tumor tissue, as tissue dynamics impede medication administration, limit immune cell infiltration, and encourage disease invasiveness.^86^, ^87^ Second, ECM stiffness disturbs endothelial cell–cell interactions, hence compromising barrier integrity and resulting in vascular leakage.^88^ Moreover, a previous study discovered that integrin β1 and its downstream effector c‐Jun N‐terminal kinase (JNK) mediates sorafenib resistance during tumor stiffening.^89^ The upregulation of PD‐L1 in cancer cells due to an increase in ECM stiffness is a tactic employed by tumor cells for adaptive immune resistance to evade immune surveillance. This upregulation may occur through the F‐actin polymerization, leading to increased expression of PD‐L1.^90^

### 2.4 ECM stiffness is involved in metabolism regulation

Tumors with a high ECM concentration have poor diffusion and low oxygen levels. The stiffness of the ECM induces the reprogramming of glucose metabolism in tumor cells, resulting in the activation of glycolysis and a decrease in tricarboxylic acid (TCA) cycle intermediates. This occurs because the exchange of molecules is hindered, hence increasing glycolytic metabolism and acidification. However, in CAFs, both glycolytic and TCA cycle intermediates were significantly enhanced when ECM stiffness was high. This implies that oxidative phosphorylation of CAFs is elevated when the ECM has a high degree of stiffness.^91^ Normal tissues control the breakdown of the glycolytic rate‐limiting enzyme phosphofructokinase (PFK) by E3 ubiquitin ligase‐containing protein 21 (TRIM21) that targets PFK. In tumor tissues, the F‐actin of the cytoskeleton forms dense bundles, which can stimulate glycolysis by binding TRIM21 and preventing it from accessing TRIM21.^26^

ECM stiffness reprograms lipid metabolism in tumor cells, increasing cell membrane fluidity, cancer invasion and metastasis. When ECM stiffness increases, mechanical signals activate integrin β1/FAK signaling through downregulation of SCD1 ubiquitin‐proteasome degradation, resulting in increased SCD1. SCD1 mediates fatty acid desaturation and increases the mono‐unsaturated fatty acids (MUFA)/saturated fatty acid (SFA) ratio. However, oleic acid, an SCD1 product, increases plasma membrane fluidity and cell invasion mediated by ECM stiffness.^32^

Mechanical stimuli coordinate the exchange of nonessential amino acids within the tumor niche. When exposed to stiff ECM, glutaminase (GLS1) and aspartate/glutamate transporter (SLC1A1) expression were increased, and Gln consumption was increased. In carcinoma cells, Gln‐derived carbon supports glutamic acid (Glu) synthesis and absorbs aspartic acid (Asp) to supply the nucleotide biosynthesis pathway. In CAFs, Gln is the main carbon source of the TCA cycle, accumulates in Asp, and absorbs Glu to supply the glutathione pathway, and the level of secreted Asp/Glu is similar to the level of Asp/Glu absorbed by cells, reaching a balance. This crosstalk process is mediated by SLC1A3.^91^ The alteration of Gln metabolism by ECM stiffness can also be manifested by microtubule glutamylation. Mechanical stress promotes tumor cell invasion by promoting MT glutamylation to stabilize MT structure.^30^ A stiff environment facilitates the regulation of creatine phosphorylation by increasing the urea cycle metabolite L‐arginine through YAP‐dependent cytoplasmic creatine kinase B‐type expression.^92^

### 2.5 ECM stiffness is involved in the promotion of angiogenesis

During tumorigenesis, ECM stiffness plays a crucial role in controlling angiogenesis.^93^ Consequently, angiogenic growth, invasion, and new vessel branching will accelerate as ECM stiffness increases.^88^ ECM stiffness upregulates MMPs activity in which membrane type 1 (MT1)‐MMP, MMP1, and MMP9 play key roles in the degradation of the ECM during angiogenesis,^94^, ^95^ thereby allowing endothelial cells to produce an increasing number of invasive angiogenic sprouts to migrate from maternal blood vessels.^96^ A previous study revealed that the inhibition of MMP significantly reduced angiogenic growth in stiffer cross‐linked gels.^88^ The increase of ECM stiffness significantly upregulates Piezo1 expression at both cell and tissue levels, and high Piezo1 expression indicates a poor prognosis in cancer patients. Piezo1 promotes the expression and secretion of vascular endothelial growth factor (VEGF), CXC chemokine ligand 16 (CXCL16), and insulin‐like growth factor binding protein 2 (IGFBP2). In addition, the upregulation/activation of Piezo1 induced by ECM stiffness inhibits the ubiquitination of HIF‐1α, which subsequently enhances the expression of downstream proangiogenic factors and accelerates angiogenesis in HCC. Furthermore, collagen 1 (COL1) enhanced tissue stiffness, leading to miR‐625‐5p expressing more Piezo1, forming positive feedback.^97^ The ECM plays a crucial role in regulating neuroblastoma (NB) angiogenesis by modulating the communication between tumor cells and endothelial cells. Surprisingly, stiffer ECMs were found to inhibit angiogenesis in NB, as they decreased the secretion of vascular endothelial growth factor isoform 165 (VEGF165). Overexpression of YAP was able to reverse this effect and restore VEGF165 secretion, suggesting that the regulation of VEGF165 by YAP may contribute to the effects of ECM stiffness on angiogenesis in NB.^98^

### 2.6 ECM stiffness is involved in the regulation of tumor immunity status

In the cancer tissue, T cell proliferation is significantly reduced on stiff ECM compared to soft ECM and spreads to a lesser extent.^99^, ^100^ The reduced number of infiltrating CD8^+^ T cells in breast tumors with high collagen density, and the significant gene expression differences induced by culture with high collagen density compared with low collagen density suggest that T cells acquire less cytotoxicity and more regulatory phenotypes.^100^ For example, mouse CD4+T cells showed higher IL‐2 secretion with increased ECM stiffness.^101^ The density and orientation of the ECM completely eliminate chemokine‐directed motility and control T‐cell localization and migration. Activated T cells actively migrate in a low‐density collagen matrix, but their migration is inhibited in dense collagen.^102^ The aligned fibers around the perivascular area and tumor epithelial cell area determine the migration trajectory of T cells and promote T cell migration through contact guidance.^103^

ECM stiffness can affect macrophage polarization and its functions in cancer.^104^ A stiff ECM induces proinflammatory hypophagocytic phenotype through positive feedback between Piezo1 and actin, YAP, and TLR4 activity,^105^, ^106^, ^107^ whereas a soft ECM induces anti‐inflammatory, hyperphagocytic phenotype of macrophages. Increased ECM stiffness in breast tumors promotes the accumulation of M2‐like macrophages.^108^ In addition, stiffness determines the migration pattern of macrophages. On soft gels, cells display Rho‐associated kinase (Rho‐kinase/ROCK)‐dependent, podosome‐independent fast amoeboid migration; on stiff gels, they adopt a ROCK‐independent, podosome‐dependent slow mesenchymal migration mode.^109^

## 3 PATHWAYS RELATED TO ECM STIFFNESS

**FIGURE 3:**
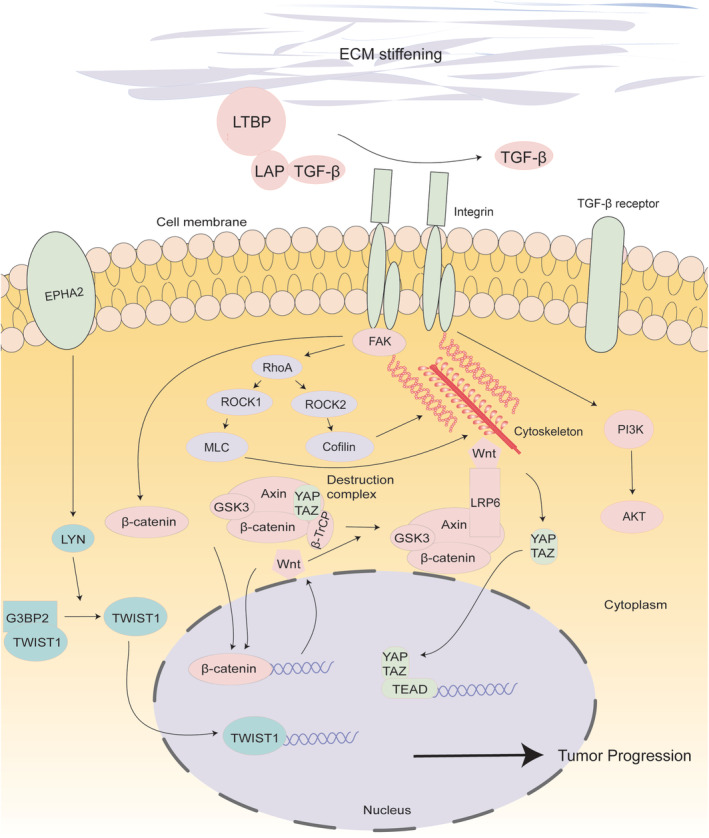
The relationship between ECM stiffness and tumor cells. The interaction among these constituents contributes to the fluctuating alterations in ECM stiffness and neoplastic growth. The depiction of genes, pathways, and transcription factors that interact with the ECM and cancer cells are outlined. FAK, focal adhesion kinase; LAP, latency‐associated peptide; LTBP, latency TGF‐β binding protein; Rho‐kinase/ROCK, Rho‐associated kinase; TAZ, Transcriptional co‐activator with PDZ‐binding motif; TEAD, TEA domain; TGF‐β1, transforming growth factor; YAP, YES‐associated protein.

Several signaling pathways have been implicated in the regulation of ECM stiffness and its impact on cellular behavior. One of the key pathways involved is the integrin‐mediated signaling pathway. Integrins are transmembrane receptors that interact with ECM components and transmit mechanical signals into the cell, leading to downstream signaling events. Activation of integrins triggers intracellular signaling cascades, including FAK and Rho GTPase signaling, which regulate cytoskeletal organization and cellular contractility. Another pathway involved in ECM stiffness‐related signaling is the Hippo pathway. The Hippo pathway plays a critical role in tissue homeostasis and organ size control. It regulates cellular proliferation and apoptosis by modulating the activity of YAP and transcriptional coactivator TAZ. ECM stiffness has been shown to activate the Hippo pathway, leading to YAP/TAZ nuclear translocation and subsequent transcriptional regulation of genes involved in cell proliferation and survival. TGF‐β signaling is also closely associated with ECM stiffness. TGF‐β is a multifunctional cytokine that regulates various cellular processes, including ECM synthesis and remodeling. It can induce the production of ECM proteins, such as collagen and fibronectin, leading to increased ECM stiffness. Moreover, TGF‐β signaling promotes the activation of myofibroblasts, which contribute to tissue fibrosis and increased ECM deposition. The main pathways involved in controlling ECM stiffness will be discussed in the following section (Figure 3).

### 3.1 YAP/TAZ signaling pathway

YAP/TAZ is a potent cell proliferation and survival regulator. It converts extracellular mechanical signals into intracellular biochemical signals and is functionally required for ECM‐stiffness‐induced mesenchymal stem cell differentiation and cell‐geometrically regulated endothelial cell survival. The YAP/TAZ pathway plays a significant role in the regulation of organ development, cell differentiation, and the self‐renewal of progenitor cells.^14^ This pathway is also responsible for an essential part of the downstream oncogenic transcriptional responses.^110^ The literature has documented two parallel modulations of YAP/TAZ: (1) the Hippo pathway, and (2) the Rho activity and actomyosin cytoskeleton pathway. The core of the Hippo signaling pathway is a kinase cascade from the tumor suppressor Hippo (Mst1 and Mst2 in mammals) to the oncoprotein Yki (YAP and TAZ in mammals).^111^ The YAP component of the Hippo signaling pathway is the major transcriptional pathway activated by mechanical stress and stiff ECM. Dupont et al. have shown that YAP and TAZ operate as intermediaries in transmitting mechanical signals from the ECM rigidity and cell shape to the nucleus. This process is dependent on the activity of Rho GTPase and the tension of the actomyosin cytoskeleton, but it does not involve the Hippo/LATS cascade.^14^ Furthermore, it has been found that YAP/TAZ play a crucial role in the differentiation of mesenchymal stem cells triggered by ECM stiffness and in the survival of endothelial cells controlled by cell geometry.^14^, ^92^ In contrast to hippo‐mediated regulation, mechanical cues may exert independent control over YAP. For example, a study of cervical cancer revealed that the peptidyl‐prolyl cis‐trans isomerase non‐mitotic alpha‐interacting protein 1 (Pin1) interacts with YAP through F‐actin, leading to positive feedback regulation of the EMT.^112^ They represent two parallel inputs converging on YAP/TAZ regulation.^113^ YAP/TAZ activity and subcellular localization are regulated by ECM stiffness, in which adhesion to stiff ECM triggers integrin aggregation, leading to signaling.^14^ Then, the YAP/TAZ protein shuttles between the nucleus and cytoplasm.^114^ Moreover, ECM stiffness affects YAP and TAZ via the Ras‐related GTPase RAP2.^115^ YAP/TAZ proteins play a relatively passive role in regulating specific signaling pathways, such as the Wnt signaling pathway in the cytoplasm. Furthermore, YAP translocate to the nucleus and binds primarily to target members of the TEA domain (TEAD) family of transcription factors to regulate the expression of proliferation‐related genes.^116^

The activation of YAP transcription factors is also a hallmark feature of CAF that promotes ECM stiffening, cancer cell invasion, and angiogenesis. In addition, YAP regulates the expression of several cytoskeletal regulators that mediate ECM remodeling, promote cancer cell invasion, including ANLN and DIAPH3, and control MYL9/MLC2 protein levels.^40^ Previous researchers identify the F‐actin‐capping/severing proteins Cofilin, CapZ, and Gelsolin as gatekeepers that limit YAP/TAZ activity in cells experiencing low mechanical stresses.^117^ High stiffness promotes the migration and proliferation of individual cells by inducing the nuclear localization of YAP/TAZ in bone metastasis‐derived cells.^78^

### 3.2 Wnt/β‐catenin signaling pathway

The Wnt/β‐catenin pathway regulates cell proliferation and differentiation and is abnormally activated during cancer progression.^118^ When stiffness rises in bone marrow mesenchymal stem cells and primary chondrocytes, mechanical activation of integrin and FAK raises the expression of β‐catenin that attaches to the Wnt promoter to upregulate its transcription, establishing positive feedback of this pathway.^119^ During tumor progression, mechanical signals drive EMT in HCC through the Wnt/β‐catenin pathway,^58^ and increased β‐catenin expression was also observed in uterine fibroids.^120^ ECM stiffness stimulates β‐catenin nuclear translocation via the matricellular protein CCN1, which promotes the cancer cell‐endothelium interaction and thus promotes cancer cell metastasis through vasculature.^121^ YAP and TAZ are components of the β‐catenin destruction complex and play biphasic roles in Wnt signaling by which the YAP/TAZ release from the complex is a tool for Wnt/β‐catenin signaling in Wnt‐ON cells.

On the other hand, YAP/TAZ is essential for β‐catenin inactivation in Wnt‐OFF cells. The competition between YAP/TAZ and LRP6 for Axin's domain is so intense that Axin's combination with YAP/TAZ is incompatible with Axin's combination with LRP6. Thus, Axin/YAP/TAZ complexes are predominant in Wnt‐OFF cells, whereas Axin/LRP6 complexes dominate in Wnt‐ON cells. Wnt signaling removes YAP/TAZ from the destruction complex, resulting in their accumulation in the nucleus and stimulation of target gene expression.^122^ Upregulation of secreted‐MMP7, a transcriptional target of Wnt‐β‐catenin signaling in the TNBC, is associated with loss of PTEN.^123^

### 3.3 PI3K/Akt/mTOR signaling pathway

PI3K/Akt/mTOR signaling is essential for many cellular biological processes, including cell growth, transfer, survival, and metabolism in all cells, including cancer.^124^ Studies have shown that ECM stiffness stimulation signal is transduced into HCC cells through integrin β1, which activates the PI3K/Akt pathway, upregulates VEGF expression,^125^ and is associated with enhanced endothelial cell proliferation, contraction, and proliferation. ECM stiffness stabilizes a mechanosensitive vinculin‐talin‐actin adhesion complex in breast cancers, allowing PI3K‐mediated phosphatidylinositol (3,4,5)‐triphosphate (PIP3) synthesis. Thus, by promoting the development of a molecular scaffold at the FA, ECM stiffness promotes oncogenic signaling via PI3K, resulting in malignant transformation.^126^ Other research elucidated the underlying changes in PI3K/Akt/mTOR signaling and found that activating this pathway by stiffer ECM was associated with increased proangiogenic secretion, proliferation, and induction of capillary‐like vessels.^127^ LOXL2 released from primary tumors causes ECM stiffness and upregulates fibronectin through the PI3K‐AKT pathway to promote lung metastasis.^128^

### 3.4 TGF‐β signaling pathway

TGF‐β is an important component of ECM that has both cancer and anticancer effects. TGF‐β can act on specific sites of ECM through the TGF‐β complex, namely latency‐associated peptide (LAP) and latency TGF‐β binding protein (LTBP) that play a critical role in increasing ECM content.^129^ Fibrotic transforming growth factor TGF‐β1 is stored in the ECM as part of large latent complexes and can be activated by integrin‐delivered cell contractile forces.^130^ In normal conditions, unstressed tissues sustained basal release of TGF‐β from local sources that is sufficient to maintain homeostasis.^131^ Growth factors directly convert cellular mechanical forces into biochemical signals. Mechanical stress‐dependent activation of TGF‐β1 is associated with the contractility and stiffness of the ECM.^132^ Recent research has found that a rise in ECM stiffness and epithelial cell contractility in pancreatic cancer might be triggered by a positive feedback loop in which TGF‐β signaling is disrupted.^133^ Endothelin‐1 or angiotensin‐II enhances myofibroblast contraction through thrombin stimulation, prompting the release of active TGF‐β1 from the ECM microenvironment.^134^ TGF‐β increased the expression of smooth muscle cell markers on stiff ECM, and Rho GTPase was also involved in marker expression.^135^

### 3.5 RhoA/Rock signaling pathway

Rho‐associated kinase (Rho‐kinase/ROCK) is an effector of the small GTPase Rho, which belongs to the protein kinase A, G, and C family (AGC) kinase family.^136^ Michael et al. disclose in their study that ROCK stimulation and subsequent actomyosin‐mediated cellular contractile force hyperproliferation, thereby increasing the burden of tumors and advancement.^137^ The RhoA/ROCK pathway is essential in cell motility and stiffness, modulating tumor progression, invasion, and metastasis. Studies have shown that overexpression of the mechanosensitive encoding of actin cross‐linking/gelling protein TAGLN in ovarian cancer can mediate ovarian cancer stiffness regulation progression through RhoA/ROCK pathway.^138^ In human salivary adenoid cystic cancer cells, ECM stiffness regulates the activity of MMPs and TIMPs, modulating their migration and invasion ability through the RhoA/ROCK pathway.^139^ Stiff ECM mediates RhoA/ROCK1/p‐MLC and RhoA/ROCK2/p‐cofilin pathways through integrin β1‐FAK activation, ultimately promoting directed migration.^140^

### 3.6 TWIST1‐G3BP2 signaling pathway

The transcription factor TWIST1 is a potent oncogene that stimulates EMT and cancer metastasis. High ECM stiffness results in ligand‐independent phosphorylation of the adrenal receptor EPHA2, which recruits and activates LYN kinase. After that, LYN phosphorylates the EMT transcription factor TWIST1,^81^ and high ECM stiffness promotes nuclear translocation of TWIST1 by releasing TWIST1 from its cytoplasmic binding partner G3BP2. Loss of G3BP2 leads to nuclear localization of constitutive TWIST1 and, in concert with increased ECM stiffness, induces EMT to promote tumor invasion and metastasis.^80^

## 4 ECM STIFFNESS MODEL

The field of tissue engineering has made substantial progress in recent years, mostly due to the creation of many sophisticated mechanical models for cell culture (Table 1). Alginate‐collagen interpenetrating network (IPN) hydrogels, which are highly adaptable and may be employed for a range of diverse purposes, are among the most often used mechanical models.^13^, ^95^, ^141^, ^142^, ^143^, ^144^ Polyacrylamide gels covered with collagen, fibronectin, or laminin to offer increased mechanical and biochemical cues are also popular.^13^, ^16^, ^19^, ^20^, ^24^, ^25^, ^29^, ^53^, ^73^, ^82^, ^84^, ^97^, ^98^, ^112^, ^121^, ^126^, ^140^, ^144^, ^145^, ^146^, ^147^, ^148^, ^149^, ^150^, ^151^, ^152^, ^153^, ^154^ Because of their mechanical flexibility and ability to simulate the elasticity of soft tissues, polydimethylsiloxane (PDMS) substrates have also been frequently used.^78^, ^155^, ^156^ Another popular option is methacrylated hyaluronic acid hydrogels, which provide a highly biocompatible environment for cell development.^157^, ^158^, ^159^, ^160^ Polyethylene glycol (PEG)‐based hydrogel scaffolds have also been intensively researched, with researchers investigating various variants and alterations to improve their properties.^161^, ^162^, ^163^, ^164^, ^165^, ^166^, ^167^ Collagen is made of gelatin and acrylate resin. Due to their capacity to imitate the mechanical characteristics of native tissue and the potential for use in tissue engineering applications, IPN hydrogels have grown in prominence.^76^, ^168^, ^169^, ^170^ The dynamic magneto‐softening hydrogel is another novel model that can modify its mechanical characteristics in response to magnetic fields. Coaxial electrospun models have also been produced, which offer a highly controllable way of creating fibrous scaffolds with variable mechanical and biological signals.^171^ For diverse tissue engineering applications, collagen gels, agarose‐collagen IPN hydrogels, alginate‐gelatin hydrogels, and reconstituted basement membrane (rBM)‐alginate IPN hydrogels have all been widely employed,^53^, ^141^, ^172^, ^173^ Chitosan‐hyaluronic acid (CHA) polyelectrolyte complex scaffolds and peptide gels offer unique mechanical and biochemical properties, making them attractive options for various tissue engineering applications.^174^, ^175^ Finally, polyisocyanide hydrogels are a relatively new addition to the mechanical model repertoire, with intriguing applications in tissue engineering and regenerative medicine.^176^ Meanwhile, the significance of co‐cultures within the 3D tumor microenvironment is pivotal. However, co‐culture environments, particularly those involving stromal cells, exhibit high complexity and can induce dynamic alterations in the physical and biochemical attributes within the tumor microenvironment. These changes encompass shifts in macrophage polarization and inflammatory states, modifications in ECM densification, and cancer cell migration, particularly influenced by the MMP and contractile activities of fibroblasts. Additionally, there is substantial ECM destruction on a large scale, particularly under the influence of transforming growth factor‐beta (TGF‐β) signaling in fibroblasts.^177^, ^178^

**TABLE 1:** A summary of the tissue engineering advancements that have led to the development of various mechanical models.

Model	Main components
Alginate‐collagen IPN hydrogels	Collagen type I, low viscosity alginic acid sodium salt stock solution, CaCl_2_
Polyacrylamide gels	Acrylamide, bis‐acrylamide, ammonium persulfate, NaOH, N,N,N′,N′‐tetramethylethylenediamine, Sulfo‐SANPAH, glutaraldehyde, dichlorodimethylsilane, hepes
PDMS	Sylgard 184
Methacrylated hyaluronic acid hydrogels	Hyaluronic acid, methacrylic anhydride, NaOH, acetone, liquid nitrogen, triethanolamine, Irgacure 2959, ethanol, poly‐D‐lysine, dichlorodimethylsilane, 1‐ethyl‐3‐(3‐dimethylaminopropyl) carbodiimide, N‐hydroxysuccinimide, type I rat tail collagen
PEG‐based hydrogel scaffolds	Poly (ethylene glycol) diacrylate L, acrylic acid, 2,2,6,6‐tetramethylpiperidine 1‐oxyl, Irgacure 2959, TINUVIN 234
gelMA‐collagen IPN hydrogels	Type‐A porcine skin gelatin, methacrylic anhydride, phenyl‐2,4,6‐trimethylbenzoylphosphinate, collagen
Dynamic magneto‐softening hydrogel	Gelatin, hyaluronic acid, glycidyl methacrylate, MMPs coated with thiol groups, permanent magnet
Coaxial electrospun model	Electrospinning, gelatin
Collagen gels	Bovine collagen type I, rat collagen type I, NaHCO_3_, DMEM
Agarose‐collagen IPN hydrogels	Agarose stock solution, collagen type I, NaOH
Alginate‐gelatin hydrogels	Sodium alginate stock solution, collagen type I
rBM‐alginate‐collagen IPN hydrogels	Matrigel, alginate, DMEM, calcium sulfate
CHA polyelectrolyte complex scaffolds	CHA, acetic acid, hyaluronic acid, ammonium hydroxide
Peptide gels	Octapeptide gelator, NaOH
Polyisocyanide hydrogels	Ni‐based catalyst, chloroform, tetrahydrofuran, diethyl ether, di‐, tri‐ and tetraethylene glycol functionalized isocyano‐(D)‐alanyl‐(L)‐alanines, 1% azide appended monomer

## 5 ECM TARGETED THERAPY

Targeting ECM stiffness may be an effective cancer treatment and medication resistance method. In addition to contributing to the formation of tumors, the stiffness and cross‐linking of the ECM hamper the diffusion of anticancer medicines and immune cells within tumors. In the meantime, it is crucial to recognize the heterogeneity of tumor cells, as various cells may exhibit distinct responses to the ECM. Disparities may emerge not only between different cell lines, even within the same cancer type, but also due to heterogeneity within a single cancer cell line.^179^ The effectiveness of drugs targeting specific cell‐ECM interactions may be compromised in the presence of tumor heterogeneity. Valuable insights into this aspect have been unveiled through 3D tissue culture models and advanced biophysical characterization studies.^180^ In the following sections, we will highlight the targeting or inhibition of specific targetable genes or pathways involved in ECM stiffness (Figure 3, Table 2).

**TABLE 2:** Emphasize the targeting or inhibition of particular genes or pathways that are susceptible to intervention and play a role in modulating ECM stiffness.

ECM components	Methods	Drugs
Collagen	Collagen Biosynthesis Inhibition	Losartan^188^
	Antifibrosis	Halofuginone
	Targeting cancer‐associated fibroblasts	CAR T cell therapy
	Disrupting collagen fiber alignment	DDR1‐neutralizing antibody
	Collagenase	Collagenase encapsulated nanoparticles and hydrogels
LOX	Irreversible inhibitors	BAPN,^192^ PXS‐5120A, PXS‐5153A, PAT‐1251, CCT365623^192^
	Monoclonal antibodies	Simtuzumab
	Copper chelator	Ammonium tetrathiomolybdate
MMPs	MMPIs	Batimastat, tanomastat, sinulariolide
	Chemically modified tetracyclines	Metastat, minocycline, doxycycline
	Off‐target inhibitors	Zoledronic acid, letrozole
	MicroRNAs	miR‐146a, miR‐146 b, miR‐93‐5p
	Monoclonal antibodies	DX‐2400, GS‐5745
TGF‐β	Antisense oligonucleotides	Trabedersen, lucanix
	Monoclonal antibodies	Fresolimumab
	Ligand traps	AVID200
	TβR kinase inhibitors	Galunisertib, vactosertib, LY3200882
	Cyclic RGD pentapeptides	Cilengitide
Integrin	Small peptide antagonists	Cilengitide,^190^ volociximab,^190^ ATN‐161

### 5.1 MMP‐targeted therapy

Even though an elevated MMP level is connected to the degradation and reconfiguration of the ECM, it is possible to target it to reduce stiffness and tumor invasiveness. In clinical studies, most treatments that target MMP activity have produced disappointing outcomes. Until now, Periostat (doxycycline hydrate, a tetracycline analogue) is the only MPI licensed in the United States for use in periodontal disease.^181^ MMP inhibitors may be more effective in early‐stage patients, and one of the major tasks in the future is to develop antibodies that are specific for a single MMP and have little cross‐reaction with other MMPs.^182^, ^183^

### 5.2 TGF‐β targeted therapy

Current preclinical or clinical therapies targeting TGF‐β include antisense molecules, anti‐TGF‐β cancer vaccines, monoclonal antibodies against TGF‐β, latent TGF‐β targeted therapy, Soluble TGF‐βRII and soluble TGF‐βRIII (betaglycan), TGF‐β receptor kinase inhibitors (TRKI).^184^ However, due to the wide distribution of TGF‐β in the whole body and the complexity of its function, anti‐TGF‐β methods still need more verification before they can be put into clinical practice.^185^ TGF‐β and PD‐L1 initiate signaling pathways with complementary, non‐redundant immunosuppressive functions in TME. The combination of PD‐L1 and TGF‐β inhibitors has been found to improve clinical outcomes and potentially reduce toxicity and is currently being tested.^186^

### 5.3 Collagen‐targeted therapy

Collagen's significant role in the construction of the TME, mechanotransduction processes, cancer progression, dissemination, and therapeutic response suggests that it could serve as a potential therapeutic target for tumors. Many anti‐tumor agents (e.g., anti‐VEGF agents) have been shown to increase blood pressure,^187^ and studies have shown that the classic antihypertensive agent angiotensin II receptor antagonist losartan reduces stromal collagen content in tumors while improving intratumoral and intravenously delivered nanoparticles (Doxil, HSV) penetration and therapeutic effect. Moreover, losartan is FDA‐approved for clinical use; it is a safe and effective adjunct to novel therapies targeting tumor collagen synthesis.^188^

### 5.4 Integrin‐targeted therapy

Many preclinical studies have shown that integrin inhibitors can significantly inhibit tumor growth and metastasis. None of the currently marketed integrin inhibitor drugs are administered orally, and none are planned for cancer therapy.^189^ Drugs for cancer treatment, such as cilengitide, an inhibitor of αvβ3 and αvβ5 integrins, and volociximab, which targets integrin α5β1, have shown promising potential in preclinical studies.^190^

### 5.5 LOX‐targeted therapy

As research on LOX continues to deepen, LOX increasingly shows its significant potential as a therapeutic target. The irreversible LOX inhibitor β‐aminopropionitrile (BAPN) reduces the metastatic colonization potential of circulating breast cancer cells.^191^ CCT365623, an oral LOX inhibitor containing aminomethylenethiophene (AMT) scaffold, showed excellent anti‐LOX potency, selectivity, pharmacokinetic properties, and anti‐metastasis effects.^192^ In contrast, experimental manipulation of ECM with an anti‐LOXL2 antibody in syngeneic orthotopic pancreatic ductal adenocarcinoma (PDA) mouse models resulted in a significant decrease in ECM content, decreased tissue stiffness, and accelerated tumor growth, resulting in decreased overall survival. This study suggests that ECM plays an essential protective function in PDA and cautions against clinically implementing ECM depletion strategies.^193^

### 5.6 Gene‐targeted therapy

Genome editing holds enormous potential for cancer therapy. Combining genome editing with tissue engineering techniques is one approach to addressing tissue stiffness in cancer. For example, researchers have used gene editing to modify the properties of the ECM, which is the network of proteins and other molecules surrounding tissue cells. By altering the composition and stiffness of the ECM, researchers have improved the delivery of therapeutic agents to cancer cells and enhanced the efficacy of cancer treatments.

Another approach is to use gene editing to modify the cancer cells' properties. For example, researchers have used genome editing to alter the expression of genes involved in cancer cell migration and invasion, which can help to prevent cancer cells from spreading to other parts of the body. According to the evidence, the combination of FAK siRNA and CRISPR‐PD‐L1‐LNP (a dendrimer lipid nanoparticle with multiple functions) was able to decrease the stiffness of the ECM and effectively suppress PD‐L1 expression using CRISPR/Cas gene editing. This approach resulted in a significant reduction in tumor growth and metastasis in four mouse models of cancer.^194^ Genetic knockout of Piezo2 in SOX2 medulloblastoma cells decreased local tissue stiffness, increased drug delivery across the blood‐tumor barrier, and improved survival.^195^

## 6 THE ECM‐TARGETED CANCER THERAPY PARADOX

ECM‐targeted cancer therapies have emerged promising strategies to disrupt the supportive microenvironment essential for tumor growth and survival. The rationale behind these therapies is to degrade or remodel the ECM components to inhibit cancer progression. However, this approach presents a paradoxical challenge that has generated significant debate among researchers and clinicians.^196^, ^197^, ^198^ On one hand, degrading the ECM can disrupt the structural and signaling support that tumors rely on, potentially impeding cancer growth.^199^ On the other hand, ECM degradation can inadvertently facilitate metastasis by creating a more permissive environment for cancer cell migration and invasion.^200^ The ECM serves as a critical barrier to tumor cell dissemination, and its degradation by proteolytic enzymes, such as MMPs, can enhance tumor invasiveness. MMPs, for instance, break down ECM components like collagen and fibronectin, releasing bioactive fragments and growth factors that promote angiogenesis, immune evasion, and the migration of cancer cells to distant sites. Overexpression of MMP‐8 in cancer cells is linked to decreased survival rates in patients with ovarian cancer and hepatocellular carcinoma, indicating its pro‐tumor effects.^201^ On the other hand, MMP‐8 also has anti‐tumor effects. Studies have demonstrated that it can limit the invasion of cancer cells in laboratory settings. Additionally, low levels of MMP‐8 expression are associated with lower survival rates in patients with oral tongue squamous cell carcinoma.^202^ Moreover, germline deletion of MMP‐8 in mice increased susceptibility to chemically induced skin tumors, whereas bone marrow transplants of MMP‐8‐expressing neutrophils restored tumor protection.^203^ The mechanical properties of the ECM, such as stiffness and density, are also altered during degradation, influencing cancer cell behavior and metastatic potential. This paradox is evident in the dual role of MMPs in cancer progression.

Similarly, therapies targeting other ECM components, such as collagen and fibronectin, must be carefully evaluated for their potential to enhance metastatic spread inadvertently. For example, a study by Lu et al. discusses how ECM degradation and remodeling play critical roles in both tumor suppression and promotion, depending on the context and extent of ECM changes.^204^ Additionally, research by Pickup et al. explores how the ECM modulates various hallmarks of cancer, including invasion and metastasis, underscoring the complexity of targeting the ECM in cancer therapy.^205^

The challenge lies in balancing the anti‐tumor benefits of ECM‐targeted therapies with the risk of promoting metastasis. Future research must focus on understanding the specific conditions under which ECM remodeling contributes to metastasis and developing strategies to mitigate these risks. This could involve combination therapies that simultaneously target ECM components and other pathways critical for metastasis or designing selective inhibitors that prevent harmful ECM degradation without disrupting its structural integrity. By addressing these complexities, it may be possible to harness the therapeutic potential of ECM‐targeted treatments while minimizing the associated risks of metastasis.

## 7 CONCLUSIONS AND PROSPECTIVES

Cancer is a complicated and multifaceted illness caused by continual interactions between cancer cells, the ECM, and other cell types in the TME. Therefore, the stiffness of the ECM plays an important role throughout the advancement of cancer, making it a prospective therapeutic target for cancer treatment. A vicious loop that causes cancer growth is characterized by the stifling and remodeling of the ECM. An increase in the ECM stiffness causes a mechanotransduction signal to be triggered, which then stimulates the production of MMP from stromal cells and cancer cells. An increase in MMP activity encourages the breakdown and rearrangement of ECM components. Therefore, the stiffness of ECM may be very dynamic in cancer. Remodeling the ECM connected to mechanotransduction is essential for activating cancer‐associated stromal cells, forming new blood vessels, evading the immune system, and the movement and invasion of tumor cells. The cytoskeletal contractility of tumor and stromal cells is affected by mechanical signals linked to the stiffness of the ECM. Although integrins and FA dynamics are significant mediators of ECM stiffening‐induced cancer growth, other functions that respond to growing ECM stiffness and permit mechanosignal transmission to all tumor components need to be found.

Meanwhile, further study is required to determine the mechanisms behind the regulation of the microenvironment of cancer, immune surveillance, and cancer dissemination by ECM stiffness. The identification of tumor stiffness may help determine the prognosis of cancer patients because of the significant roles that ECM stiffness plays in the evolution of the disease. Elastography techniques such as shear wave elastography, magnetic resonance elastography, and transient elastography may all be used to evaluate tissue stiffness in a non‐invasive manner. The biophysical effects of cancer's ECM stiffness may interfere with medication transport and the susceptibility of cancer cells to anticancer treatments. Consequently, identifying tumor stiffness may assist in the patient stratification process before treatment. Collagenase has the ability to directly deplete collagen, which in turn lowers the stiffness of the ECM and increases medication penetration and sensitivity in tumors. On the other hand, there are worries about the treatment's side effects when administered systemically. The production and assembly of collagen may be inhibited, which is an alternate treatment method. YAP/TAZ inhibitors, TGF‐β inhibitors, and integrin inhibitors may inhibit collagen production. Piezo, integrin, and YAP/TAZ inhibitors can inhibit collagen production and deposition, as well as the mechanotransduction that results from the stiffness of the ECM. Clinical studies have already tested a few of these possible treatment pathways in human patients. Although the outcomes of clinical studies using integrins inhibitors have been mostly discouraging, we still hope that other pipelines, such as those involving the Hippo/YAP pathway inhibitors, may provide more promising findings. Because of the many functions that ECM plays in the course of cancer and the fluid nature of ECM remodeling, it is essential to emphasize that there may be a great deal of resistance and difficulty in targeting ECM stiffness in cancer.

In brief, the ECM significantly contributes to the microenvironment of almost every cell in the human body, and abnormalities in its control are strongly linked to the onset and progression of a wide variety of illnesses, including cancer. In the last few years, there have been a lot of advancements made regarding the effective use of our understanding of ECM dysregulation in the design of anticancer treatment, and there have been a lot of accomplishments. In this article, we summarized the mechanisms underlying the interplay between stromal mechanisms and tumorigenesis and progression, as well as current therapeutic strategies targeting different components of ECM. The mechanism of action of ECM mechanics has been shown to play an important role in cancer progression. This article introduces the clinical application and potential drug targets of drugs in cancer treatment. Drugs targeting the mechanical components of the ECM hold promise for cancer treatment, but many treatments are still in preclinical or clinical trials awaiting further validation by scientists.

## AUTHOR CONTRIBUTIONS

**Ximo Zhang:** Conceptualization; data curation; formal analysis; methodology; writing – original draft. **Abdullah Al‐Danakh:** Conceptualization; writing – original draft; writing – review and editing. **Xinqing Zhu:** Methodology; resources; visualization. **Dan Feng:** Conceptualization; investigation; writing – original draft. **Linlin Yang:** Data curation; resources; visualization; writing – original draft. **Haotian Wu:** Conceptualization; data curation; writing – original draft. **Yingying Li:** Visualization; writing – review and editing. **Shujing Wang:** Supervision; writing – review and editing. **Qiwei Chen:** Conceptualization; funding acquisition; methodology; validation; writing – review and editing. **Deyong Yang:** Project administration; writing – review and editing.

## FUNDING INFORMATION

This work was supported by the National Natural Science Foundation of China (82203730, 32271336), and Dalian Medical University, Interdisciplinary Research Cooperation Project Team Funding (JCHZ2023021).

## CONFLICT OF INTEREST STATEMENT

The authors declare no conflicts of interest.

### PEER REVIEW

The peer review history for this article is available at https://www.webofscience.com/api/gateway/wos/peer-review/10.1002/btm2.10698.

## Data Availability

Data sharing not applicable to this article as no datasets were generated or analysed during the current study.
